# Response to Placebo in Fragile X Syndrome Clinical Trials: An Initial Analysis

**DOI:** 10.3390/brainsci10090629

**Published:** 2020-09-11

**Authors:** Skylar Luu, Haley Province, Elizabeth Berry-Kravis, Randi Hagerman, David Hessl, Dhananjay Vaidya, Reymundo Lozano, Hilary Rosselot, Craig Erickson, Walter E. Kaufmann, Dejan B. Budimirovic

**Affiliations:** 1 Albany Medical Center, Albany Medical College, 43 New Scotland Ave, Albany, NY 12208, USA; luus1@amc.edu; 2 Feinberg School of Medicine, Northwestern University, 420 E. Superior St, Chicago, IL 60611, USA; haley.province@northwestern.edu; 3 Departments of Pediatrics, Neurological Sciences, Biochemistry, Rush University Medical Center, 1725 West Harrison, Suite 718, Chicago, IL 60612, USA; elizabeth_berry-kravis@rush.edu; 4 MIND Institute and the Department of Pediatrics, University of California Davis Medical Center, 2825 50th Street, Sacramento, CA 95817, USA; rjhagerman@ucdavis.edu; 5 MIND Institute and the Department of Psychiatry and Behavioral Sciences, University of California Davis Medical Center, 2825 50th Street, Sacramento, CA 95817, USA; drhessl@ucdavis.edu; 6 Department of Medicine, Johns Hopkins University School of Medicine, Baltimore, MD 21218, USA; dvaidya1@jhmi.edu; 7 Departments of Genetics and Genomic Sciences, Pediatrics and Psychiatry, Icahn School of Medicine at Mount Sinai, 1 Gustave L. Levy Pl, New York, NY 10029, USA; Reymundo.lozano@mssm.edu; 8 National Fragile X Foundation, McLean, VA 22102, USA; hilary@fragilex.org; 9 Division of Child and Adolescent Psychiatry, Cincinnati Children’s Hospital Medical Center and the University of Cincinnati College of Medicine, 3333 Burnet Avenue, MLC 4002, Cincinnati, OH 45229, USA; Craig.Erickson@cchmc.org; 10 Boston Children’s Hospital and Department of Human Genetics, Emory University School of Medicine, 615 Michael Street, Atlanta, GA 30322, USA; 11 Kennedy Krieger Institute/The Johns Hopkins Medical Institutions, Department of Psychiatry & Behavioral Sciences-Child Psychiatry, the Johns Hopkins University School of Medicine, 1741 Ashland Ave, Rm 241, Baltimore, MD 21205, USA

**Keywords:** fragile X syndrome, clinical trials placebo effect, meta-analysis

## Abstract

Fragile X syndrome (FXS) is the leading cause of inherited intellectual disability and autism spectrum disorder. Individuals with FXS often present with a wide range of cognitive deficits and problem behaviors. Educational, behavioral and pharmacological interventions are used to manage these and other complex issues affecting individuals with FXS. Despite the success of preclinical models and early-phase drug clinical studies in FXS, large-scale randomized-controlled trials have failed to meet primary endpoints. Currently, no targeted or disease-modifying treatments for FXS have received regulatory approval. Here, we examined the placebo response in FXS clinical trials conducted between 2006 and 2018. Specifically, we performed a meta-analysis of placebo-treated groups in eight double-blind, randomized controlled trials. Placebo groups demonstrated significant improvements on caregiver-rated efficacy endpoints, which were greater in adolescents and adults than in children. Among the latter measures, the Visual Analog Scale scores displayed the greatest improvements, whereas the positive effects on the Vineland-II Adaptive Behavior Composite and the Aberrant Behavior Checklist-Community/fragile X version were statistically significant in both children and adolescents/adults. Although the Clinical Global Impression scale Improvement appears to have exhibited a substantial placebo effect in multiple clinical trials in FXS, limited data availability for meta-analysis, prevented us from drawing conclusions. No placebo-related improvements were observed in performance-rated measures. These findings raise substantial concerns about placebo effects in outcome measures commonly used in the randomized-controlled trials in FXS and suggest several potential improvements in the study design and implementation of such trials. Considering the small number of trials available for this study, larger and more detailed follow up meta-analyses are needed. Meanwhile, efforts to improve the measurement properties of endpoints and rater training in drug trials in FXS should be prioritized.

## 1. Introduction

Fragile X syndrome (FXS) is the most common cause of inherited intellectual disability (ID) and single-gene disorder associated with autism spectrum disorder (ASD) [1,2,3,4]. FXS is caused by a CGG-repeat expansion in the 5’ untranslated region of the Fragile X Mental Retardation 1 (*FMR1*) gene (full mutation-FM, >200 CGGs). The FM triggers epigenetic silencing (hyper-methylation) of the gene, which results in marked reduction of the *FMR1* product (Fragile X Mental Retardation Protein, FMRP). FMRP is an RNA-binding protein that regulates the translation of ~4% of the brain’s mRNA, including many transcripts from genes implicated in ASD [5,6], and serves as a critical regulatory protein for developmental and ongoing synaptic plasticity. The FMRP expression in the brain is the ultimate factor determining the severity of the neurobehavioral phenotype. Data integration that enables clarification of the relationships between the *FMR1* genotype and FMRP and the neurobehavioral phenotype, including ASD, continue to grow [7,8].

Individuals with FXS present with cognitive impairment and a wide range of problem behaviors, such as ASD, general and social anxiety, attentional network deficits, repetitive and perseverative behaviors, and sensory over-reactivity [1,2,3,4]. About 40–50% of males and 20% of females meet the criteria for ASD [2]. In addition to adequate educational placements and behavioral interventions, these problem behaviors often require a combination of complex, symptom-based pharmacological interventions [1,2,3,4]. To date, no target-symptom approaches or disease-modifying treatments for FXS have received regulatory approval [7]. 

Animal and in vitro FXS models have led to great progress in drug identification and clinical trials in humans with FXS. Nevertheless, translating preclinical successes targeting core phenotypes associated with FXS into clinical trials has been a challenge [7,8]. At present, no definitive large-scale placebo-controlled trial has met primary endpoints [8,9]. Lacking precedent, the ‘first-wave’ of FXS clinical trials was felt to reflect the problems inherent in “building the bridge while crossing it.” All these large-scale and well-powered trials failed, suggesting issues with the predictive value of animal models, adequacy of trial design, age of treated cohorts, and, to some extent, outcome measure selection [7,9]. The ‘lessons learned’ have, therefore, prompted efforts at developing and validating improved outcome measures [7,8,9,10]. However, less attention has been given to other critical factors that could have negatively impacted these failed trials. One of the most notable is the placebo effect, which likely played a role due to the trials heavily depending on parent/caregiver reporting [11,12].

Placebo response has steadily increased over time in neurologic and, in particular, psychiatric randomized controlled trials (RCTs), potentially obscuring any improvements due to drug treatment [12,13,14,15,16]. Preliminary studies in pediatric populations with idiopathic ASD have found increased placebo response associated with the use of clinician- over caregiver-rated outcome measures [16]. RCTs in FXS have mainly relied on subjective, apparently “placebo-sensitive” clinician- and caregiver-rated outcome measures due to the lack of well-validated, reliable, linked to a clinical outcome, and sensitive to change biomarkers [9]. The use of direct performance-rated cognitive and achievement testing in clinical trials has proven difficult in FXS. While such tests are used for clinical assessments, there are substantial floor effects in many tests not designed for or validated in populations with ID [17]. These tests lack short-term sensitivity and, in some cases, there is a need to administer different levels of tests to capture the entire range of function in the individual or disorder. This is an area of ongoing intensive work, and tests that can circumvent floor effects are being validated [18]. These tests show good test–retest reproducibility in ID populations, including FXS, covering a broad range of function; tests such as expressive language sampling (ELS) [19] and NIH Toolbox [20], that are expected to be sensitive to short-term change, are in development for trials in FXS [8,9]. Recent advances in molecular-phenotype relationships underscore links between *FMR1* expansion, gene methylation, FMRP deficit and overall severity of neurobehavioral phenotypes [21,22]. Despite these concerns, the extent to which the placebo effect has impacted FXS trial outcomes in general or specific outcome measures is unknown [9].

Here, we present the first meta-analysis of RCTs in FXS with a focus on the placebo effect response. Rather than evaluating the impact of the placebo effect on the overall outcome of these trials, we have focused on examining whether the placebo groups showed significant improvements in eight RCTs of individuals with FXS conducted between 2006 and 2018. We aimed at answering the following questions: 

Did placebo groups show significant and clinically meaningful responses?

Were clinician- and caregiver-rated outcome measures equally affected by placebo response?

Did age influence response in placebo groups?

## 2. Materials and Methods

### 2.1. Clinical Trials

Eight double-blind RCTs, conducted between 2002–2015 and published in English between 2006 and 2018 [23,24,25,26,27,28,29,30] were identified through a search of clinicaltrials.gov (https://clinicaltrials.gov) and personal communications with other investigators. Most of these FXS trials evaluated compounds that modulate either glutamatergic or GABAergic transmission, which were analyzed in this study and described in Table S1 [9].

### 2.2. Outcome Measures

Clinician-rated outcome measures include ratings of clinician observations and standardized performance-rated instruments. The first type included the Clinician Global Impression scale-Improvement (CGI-I), which is predominantly informed by caregiver reporting but administered and rated by the clinician in order to best determine the participant’s overall condition. Performance-rated measures are direct assessments of a participant’s skills captured by a trained examiner. These measures are thought to capture a more valid and direct evaluation of the participant’s functionality as the scoring is based on the participant’s performance of specific assessment tasks. Participants’ active effort in the assessment tasks directly impacts their outcome [31,32]. The most common performance-rated measures used in the FXS studies were: the Mullen Scales of Early Learning, Composite (MSEL Comp); the Autism Diagnostic Observation Schedule (ADOS); Preschool Language Scale 5th Ed, including Composite (PLS-5 Comp), Auditory Comprehension raw score (PLS-5_ac_rs), and Expressive Communication raw score (PLS-5_ec_rs); the *Repeatable Battery for the Assessment of Neuropsychological Status* (*RBANS)* Memory 10 subtests; and the Test of Attentional Performance for Children (Testbatterie zur Aufmerksamkeitsprüfung für Kinder; KiTAP) alertness, distractibility, flexibility and go/no-go subtests. 

Caregiver-rated outcome measures are informed by caregiver report and observation of the participant. For an individual to qualify as an acceptable caregiver rater, they typically need to meet certain requirements specific to the length of time knowing the participant or spending with the participant. The most common caregiver-rated outcome measure used in the FXS studies was the Aberrant Behavior Checklist-Community (ABC-C), scored using both the original scoring and fragile X (ABC-CFX)-specific factor structure scoring [33]. The trials included both total and subscale scores (Irritability (Irr), Social Unresponsiveness/Lethargy/(SU/L), Stereotypy (Stereo), Hyperactivity (Hyper), Inappropriate Speech (Speech) and Social Avoidance (SA) [23,25,26]. Other caregiver-rated outcome measures included the Visual Analog Scale composite (VAS Comp) and anxiety/disruptive (VAS Anx and Dis) subscales, as well as the Sensory Processing Measure-Preschool Social Participation subtest (SPM-P Soci Part), the Anxiety Depression and Mood Scale (ADAMS) total score, the Social Responsiveness Scale™ (SRS), the Repetitive Behavior Scale-Revised (RBS-R), and the Vineland Adaptive Behavior Scales-II Composite (Vineland-II Comp) and Maladaptive Behavior scale (Vineland-II Mal) [34].

It is important to note that the aforementioned outcome measures were classified using the previously stated criteria for the purposes of the meta-analysis. Some of these outcome measures have been categorized differently across trials given the overlap between caregiver- and clinician-rated input, specifically the CGI-I and Vineland-II [9]. The Vineland-II has been previously classified in both categories depending on the definitions of the given study. The CGI-I is consistently considered a clinician-rated measure; yet, in behavioral studies, it actually serves as a caregiver-informed report of the participant’s behavior, administered and rated by the clinician in a very structured way. In contrast, ABC-C and ABC-CFX are purely caregiver-rated measures. While these measures are divided into categories, they comprise a spectrum of structure in administration with both caregiver and clinician input incorporated into the rating. The clinician input in these measures ranges from CGI-I > Vineland-II > ABC-C and ABC-CFX, with caregiver input clearly informing the rating of each measure. 

### 2.3. Data Extraction

Study information such as specific treatment, sample size, participant age range, trial phase, duration, primary and secondary outcome measures and details about design were recorded in an Excel spreadsheet. In a separate spreadsheet, baseline and End-of-Treatment (EOT) data for primary and secondary efficacy endpoints in placebo groups were extracted independently by H.P., and S.L. Differences (delta) between EOT and baseline for outcome measures were extracted directly from the original publication, when available, or calculated from baseline and EOT values. Raw data were requested from authors if published data were reported in a format other than descriptive values (i.e., mean, standard deviation, standard error of mean). Outcome measures used in a single study did not qualify for the meta-analyses; however, data were displayed in Table 1 to provide context and assist in meta-analyses’ data interpretation.

### 2.4. Data Analysis

Analyses included descriptive statistics, such as calculations of proportions, for dataset characterization and meta-analyses. Data from groups receiving active drug were not included in the meta-analyses since the focus of the analyses was whether placebo per se led to improvement in outcome measures not its overall contribution to efficacy. Thus, meta-analyses included score differences (deltas) between EOT and baseline time points in the placebo groups as primary variable efficacy endpoint. If these differences were not provided, baseline and EOT scores were extracted and the difference calculated. In these instances, a previously applied pre-post correlation of 0.7 was assumed to estimate the standard error of the difference [16]. As sensitivity analyses, correlations of 0 and 0.5 were also applied. For efficacy endpoints used in more than one study, I^2^ and its corresponding *p*-value were calculated as a measure (percentage) of variance that is attributable to heterogeneity between studies [13]. Efficacy endpoints with non-significant heterogeneity (I^2^ *p* > 0.05), were analyzed by fixed effects meta-analyses. For those efficacy endpoints showing significant heterogeneity (I^2^ *p* ≤ 0.05), random effects meta-analyses were carried out. Other aspects of the meta-analyses’ procedure have been previously reported [16,35]. 

To determine the functional or clinical significance of the improvements in placebo groups, the effect size of delta scores was calculated as a function of SDs at baseline. In addition, for performance-rated outcome measures, we compared differences between baseline and EOT scores with expected developmental changes (either monthly or annually) for the endpoint as estimated by natural history studies (e.g., Vineland-II [35]). For measures with psychometric reference data (e.g., ABC-CFX [33]), we compared the delta scores with the SD/mean ratio for individuals in a specific age range.

Meta-analyses were performed using the R “meta” package [36,37], while other statistical analyses were done using SPSS version 25 (IBM, Armonk, NY, USA).

## 3. Results

### 3.1. Significant Improvements Detected in Groups Receiving Placebo Treatment

Table 1 and Table 2 reveal that most endpoints used in FXS drug trials have been applied to a single study. Among clinician- and performance-rated outcome measures, only the CGI-I was employed in more than one trial and therefore subjected to this meta-analysis [9]. As a category, caregiver-rated outcome measures were an exception as a wide array was applied across several studies. These instruments included the VAS and multiple subscales of the ABC-C and ABC-CFX. Table 1 depicts the outcome measures included in this meta-analysis as well as the instruments utilized in other trials to provide an overview of endpoint changes in FXS drug trials. 

The table includes mean score differences between baseline and EOT in placebo-treated groups, 95% confidence intervals (CIs), heterogeneity indices among studies in the meta-analysis, and the type of employed meta-analysis. Score differences and CIs were calculated using an estimated pre-post correlation of 0.7 [16]. 

The clinician-rated outcome measures used in these trials were the same across the two age groups (children, age <12 years old; adolescents/adults, age ≥12 years old). Mean changes in CGI-I scores after placebo treatment were non-significant in a total seven studies of either children (3/7, *p* = 0.56) or adolescents/adults (4/7, *p* = 0.69). In contrast, Table 2 shows a substantial placebo effect at the end of the study on the CGI-I in these trials (mean CGI-I ranged 2.6–3.5 across nine combined children and adolescents/adults trials [23,24,25,26,27,28,29,30,38] with <4 representing improvement whereas 4 no change, and in most cases SEMs not overlapping 4).

Since the limited data analyzed here is not representative, and given the contradiction with previous trial findings, conclusions cannot be drawn about this widely used clinician measure. As for the former, half (4/8) of the studies had available CGI data [23,26,27,28], which included only one out of three large-scale, well-powered studies [26]. Out of the four studies that had CGI data, three of them included children [23,27,28] and three included adolescents/adults [23,26,27].

All performance-rated outcome measures used in the eight FXS drug trials were applied to a single study, either of children or adolescent/adults. No placebo improvements were noted in the analysis of these measures. While not included in the meta-analyses, it is important to note that only the PLS-5 Comp showed borderline significant changes in scores.

Changes in caregiver-rated efficacy endpoints differed between trials depending on the participants’ age. The Vineland-II Comp score significantly improved in six studies of both children (2/6, *p* = 0.012) and adolescent/adults (4/6, *p* = 0.043). The VAS changed in two studies involving children (*p* < 0.001). Changes in total and subscales scores of the ABC-C and the ABC-CFX were variable, and most studies used the latter FXS-revised version [33]. ABC-CFX Total scores (2 studies, *p* < 0.001), and on the ABC-CFX Stereo (2 studies, *p* < 0.001) and ABC-CFX Hyper (2 studies, *p* = 0.002) subscales improved significantly only in trials of older participants. In contrast, comparisons of delta scores on the ABC-CFX SA showed age-dependent changes; while there were marked improvements in adolescent/adult trials (three studies; *p* < 0.001), there were no significant changes in trials with children (two studies; *p* = 0.28). The two original ABC-C subscales revealed no improvements in seven studies: the Irr (3/7 in children, *p* = 0.15; 4/7 in adolescents/adults, *p* = 0.22) and the SU/L (3/7 in children, *p* = 0.38; 4/7 in adolescents/adults, *p* = 0.15). 

### 3.2. Sensitivity Analyses Confirmed Significant Improvement in Placebo Groups

Complementary sensitivity analyses using estimated pre-post correlation of 0 or 0.5, illustrated in Table 3 for correlations of 0.5, demonstrated almost identical results to those summarized in the preceding section. Performance-rated sensitivity analysis could not be calculated as it was only used in one study.

### 3.3. Older Participants Tended to Show Greater Improvements with Placebo Treatment

Table 4 depicts outcome measures with significant improvements in children, which were independent of the estimated pre-post correlations. Two caregiver-rated efficacy endpoints, the Vineland-II Comp score and the VAS, met these criteria. 

Table 5 presents data for trials involving adolescents and adults, showing that the Vineland-II Comp scores also improved in older individuals. In addition, as mentioned above in Section 3.1, total scores on the ABC-C_FX_ and for three of its subscales (ABC-C_FX_ Hyper, SA, Stereo) also improved significantly. 

To determine the influence of participants’ age on improvements in response to treatment with placebo, we examined the portion of studies in each age group showing positive effects on caregiver-rated measures. There were no sufficient clinician- or performance-rated endpoints for similar analyses. Since multiple ABC-CFX and ABC-C scores were used in the trials, only subscales were included in the analysis to avoid redundancy. Regardless of the applied pre-post correlation, two of the five subscales (40%) improved in children while five of the seven (71%) improved in adolescents/adults. These observations suggest that caregivers of older individuals with FXS tend to report positive responses to placebo more frequently.

### 3.4. Improvements in Placebo Groups Are Substantial and Functionally Meaningful

To determine whether the statistically significant improvements in placebo groups represented functionally meaningful changes, we evaluated several parameters. The first was the effect size of these improvements; considering that one unit of standard deviation of a cognitive or behavioral measure corresponds approximately to a Cohen’s *d* of 1.0 [39,40], we measured the proportion of effect size represented by each mean score change (Table 6). 

If available, effect size was complemented by comparisons with reference values for endpoints. In the case of the Vineland-II Comp, natural history data for FXS have been published for both genders and a wide age range. The latter allowed for calculations of estimated changes in standard scores per year. For the ABC-C_FX_, effect sizes were complemented by gender- and age group-based mean scores and SDs. These parameters lead to the calculation of “corrected” effect sizes, based on comparable populations rather than the cohorts under study.

In terms of effect size, score changes in the ABC-C_FX_ and the VAS corresponded to small to medium and large to very large score change ranges, respectively. Improvements in the Vineland-II Comp were smaller, at the negligible effect size level. Nonetheless, the reported increases in its standard scores are in contrast with the natural evolution of a decrease in adaptive behavior scores in FXS [41]. Males with FXS between ages of 2–12 years show an average annual decrease of approximately 2.49 units of standard scores [41]. In contrast, we estimated 1.71 units of standard score improvements in placebo groups in studies with durations between one and six months. Problem behavior rating scales, such as the ABC-C_FX_, cannot be analyzed over time like the Vineland-II Comp [9]. Nonetheless, published reference values [33] for ABC-C_FX_ Total or subscale scores show that in males older than 12 years, SDs tend to be large with respect to mean scores (80–110% mean range). The mean decrease of 8.6 total score reported here is equivalent to 31% of the baseline SD. Since in the present study at baseline the SD of the ABC-C_FX_ represented only 63% of the mean score, a more accurate estimate of the effect size could be based on the mean score. In this case, the ABC-C_FX_ score change would correspond to a “corrected” effect size of 0.19 (small range).

## 4. Discussion

The present study is, to our knowledge, the first meta-analysis demonstrating positive responses in participants with FXS receiving placebo in multiple drug trials. We identified significant improvements in several caregiver-rated outcome measures, but not in the single clinician-rated endpoint under evaluation. No performance-based measure was used in more than one study, making this type of endpoint not suitable for meta-analyses. Improvements in caregiver-rated outcome measures were detected in studies involving either children or adolescents/adults, with a larger proportion of the latter showing a positive outcome. In terms of the magnitude of improvements, as measured by effect size or reference values, two out of three outcome measures showed functionally significant changes. These data are in line with reports of increasing placebo responses in RCTs of neurologic and psychiatric disorders, and raise concerns about study design and conduct of drug trials in FXS and other neurodevelopmental disorders [11,12,14,16,42].

The main goals of this meta-analysis were to determine whether placebo-treated patients with FXS show improvements and, if so, whether positive responses were influenced by age of the participant and type of outcome measure. Multiple endpoints demonstrated improvements in placebo groups even in the face of effect heterogeneity in some trials (i.e., significant I^2^) and were strong amidst assumptions regarding pre-post correlations (i.e., 0, 0.5, 0.7). As the studies used predominantly caregiver-rated endpoints, we could not evaluate the effect of the type of measure on outcomes. The fact that the CGI-I did not reach statistical significance, contrary to a study reporting on placebo response in pediatric ASD [15], may be a random finding or reflect greater weight of caregiver input into CGI scores in autism trials. However, the CGI-1 meta-analysis data here is likely too limited to be informative as many of the prior trials in FXS appears to have shown a substantial placebo effect in the CGI-I scores at the end of the study (Table 2) [23,24,25,26,27,28,29,30,38,43].

We could not derive conclusions from performance-based measures because they were not included in the meta-analysis due to their single study use. Nonetheless, the studies included in Table 1 indicate that these endpoints do not change substantially in response to placebo administration. Overall, a larger proportion of caregiver-rated scales demonstrated improvement in older individuals with FXS treated with placebo. This is in line with the abovementioned meta-analysis of placebo effect in pediatric ASD, which reported an improvement in 60% of caregiver-rated outcome measures in adults versus approximately 25% in children [16]. Nevertheless, meta-analyses of RCTs in other treatment areas (e.g., genetically determined intellectual disability, drug resistant partial epilepsy) demonstrate a different profile with greater placebo responses in younger participants [42,44]. A recent review of best practices in FXS clinical trials also concluded that inclusion of cohorts of younger participants would reduce the placebo effect [8]. 

It is important to note not only the range of caregiver-rated outcome measures showing placebo responses (e.g., Vineland-II Comp, VAS Comp, and ABC-CFX subscales), but also the magnitude of the change in the reported values of these outcome measures. Our analyses of effect size showed that in children with FXS, the VAS Comp score improved at a very large level (i.e., 1.05–1.06 SD). As a reference, a minimal clinically important difference (MCID) is a metrics equivalent to ≥0.5 SD [39,40], which has been recently used to determine meaningful changes in adaptive behavior in ASD [45]. Although the effect size of Vineland-II Comp score changes in children was negligible, the small increases represent an opposite direction to the naturally occurring decrease in standard scores [41]. These data emphasize the potential large magnitude of responses to placebo administration in individuals with FXS and the importance of identifying and minimizing them.

The present meta-analysis shows that patients with FXS receiving placebo in drug trials can demonstrate statistically significant and clinically relevant improvements. The reporter must believe there are actual improvements in behavioral symptoms or cognition of the participant for these improvements to be captured in the outcome measures. This is important, as positive changes subjectively reported by caregivers could be due to caregiver expectations of treatment efficacy. As such, even measures like the CGI, which is clinician-rated but by and large caregiver informed in behavioral studies, appears to have captured a substantial placebo response in the recently failed clinical trials in FXS [23,24,25,26,27,28,29,30,38,43]. Thus, while our clinician-rated CGI-I meta-analysis data did not reach statistical significance due to limited available data, visual inspection of CGI-I data across multiple trials reveals the notable placebo effect on this measure. Our data also raises specific concerns about the use of caregiver-rated measures, in particular the VAS, in future FXS studies. Our findings are consistent with the suggestion that placebo responses have a greater impact on trials of older individuals with FXS [8]. Our results have other implications, including the need for improvement of currently available endpoints, decreased dependence on caregiver-rated instruments, enhanced rater training to mitigate placebo response, enrollment of younger participants and utilization of study designs directly addressing the placebo effect (e.g., placebo run-ins, adaptive designs with enrichment in non-placebo responders) [8,46]. 

## 5. Conclusions

The present study shows that patients with FXS receiving placebo demonstrate significant improvements on caregiver-rated efficacy endpoints, which were greater in adolescents and adults than in children. Among the latter measures, the VAS scores displayed the greatest improvements, while the positive effects on the Vineland-II Comp and the ABC-CFX were statistically significant in both children and adolescents/adults. Although the CGI-I appears to have exhibited a substantial placebo effect in multiple clinical trials in FXS, limited data availability for meta-analysis prevented us from drawing conclusions about this widely used clinician measure. No placebo-related improvements were observed in performance-rated measures. These findings raise substantial concerns about placebo effects in outcome measures commonly used in randomized-controlled trials in FXS.

## 6. Limitations and Future Directions 

The present study of a heterogeneous group of clinical trials in FXS focused on improvements in groups assigned to placebo, rather than a direct assessment of placebo (versus drug) responses in these trials. Moreover, it could only arrive to conclusions regarding caregiver-rated measures because of the small number of studies using other types of endpoints. Future analyses should also attempt to differentiate objective from subjective improvements in individuals with FXS. It is also important to appreciate differences between actual placebo-related improvements in behavior/cognition due to increased attention during the trial from positive changes subjectively reported by caregivers (e.g., due to their expectations of treatment efficacy). Considering these and other limitations of this study, larger follow up meta-analyses are needed. Developmental placebo response in longer (≥6 month) studies where the child grows and improves need to be considered as well [28]. Moreover, the developmental gain of the “placebo” effect observed at this young age may also enable an additive synergy to the drug effect. Thus, the placebo effect is a major and increasing challenge for trials of neurologic and psychiatric disorders of all ages [11,12]. The issues with using caregiver-rated endpoints emphasize a compelling need to study biomarkers in FXS. Neurobiology in FXS is known to be impacted early in infancy. For example, visual perceptual problems, a common deficit observed in FXS that appears amenable to the drug treatment [28], can be detected as early as the first year of life through eye-tracking studies of babies with FXS [47], throughout the lifespan. MRI can be used over a large age range in FXS, and while MRI changes will be long term, it may not be sensitive enough to capture change in shorter trials [9]. This is of relevance as the effects of fragile X gene expression on the early development of white matter structural connectivity are well established at six months of age [48]. Electroencephalography (EEG) as a potential neural biomarker of changes due to treatment has the ability to examine features of fragile X due to scalability and reproducibility and translation from mouse model to clinical intervention such as neural hyperexcitability in FXS [8,49,50]. Fragile X gene–protein biomarker development has also advanced as recent molecular phenotype studies underscore link between *FMR1* expansion, gene methylation, and FMRP deficit, and overall severity of neurobehavioral phenotype [7,8]. Newer performance-rated measures such as ELS and NIH Toolbox may be able to capture real change, controlling for placebo effect given the lack of caregiver rating [8,19,20,51]. As noted above, there are also newer trial designs that aim to limit or account for placebo response, including placebo lead-ins and adaptive trials designs [8,46]. Overall, the development of valid, sensitive-to-treatment biomarkers is necessary to reliably track treatment changes in the unfolding wave of clinical trials in FXS [8,52], which will substantially reduce the placebo effect. The examination of factors contributing to responses in placebo groups should be a continuous process [35,53] parallel to efforts at improving the measurement properties of endpoints in drug trials, including performance-rated measures [9].

## Figures and Tables

**Table 1:** Outcome measures changes in placebo-treated groups with FXS in failed clinical trials – Main Analysis.

Endpoint	of Studies	Heterogeneity (I^2^)	Heterogeneity *p-*Value	Meta-Analysis Type	Mean Score Change	95% CI		Change *p*-Value
						Lower	Upper	
**Clinician Rated ^‡^**								
CGI-I (<12 years)	3	0.97	0.00	Random	−0.45	−2.00	1.10	0.570
CGI-I (>12 years)	4	1.00	0.00	Random	0.46	−1.83	2.76	0.693
**Caregiver Rated ^‡^**								
Vineland-II Comp (<12 years)	2	0.00	0.47	Fixed	1.71	0.38	3.05	0.012 *
ABC-C_FX_ Irr (<12 years)	3	0.85	0.00	Random	−3.51	−8.29	1.26	0.149
ABC-C_FX_ SU/L (<12 years)	2	0.97	0.00	Random	−2.28	−7.41	2.85	0.383
ABC-C_FX_ SA (<12 years)	2	0.98	0.00	Random	−1.44	−4.07	1.20	0.284
VAS Comp (<12 years)	2	0.00	0.42	Fixed	1.56	0.94	2.18	<0.001 *
Vineland-II Comp (>12 years)	4	0.00	0.44	Fixed	1.74	0.08	3.39	0.040 *
ABC-C_FX_ Total (>12 years)	2	0.00	0.72	Fixed	−8.60	−12.45	−4.76	<0.001 *
ABC-C_FX_ Irr (>12 years) **	4	0.91	0.00	Random	−1.96	−5.11	1.19	0.222
ABC-C_FX_ SU/L (>12 years) **	4	0.93	0.00	Random	−1.90	−4.51	0.71	0.154
ABC-C_FX_ Stereo (>12 years)	2	0.00	0.69	Fixed	−1.04	−1.58	−0.50	<0.001 *
ABC-C_FX_ Hyper (>12 years)	2	0.00	0.84	Fixed	−1.37	−2.26	−0.49	0.002 *
ABC-C_FX_ SA (>12 years)	3	0.79	0.01	Random	−1.65	−2.61	−0.68	0.001 *
**Caregiver Rated ^†^**								
Vineland-II Mal (<12 years)	1	NA	NA	NA	−0.70	−1.05	−0.35	<0.001 *
VAS Anx and Dis (<12 years)	1	NA	NA	NA	−15.50	−22.12	−8.88	<0.001 *
ABC-C_FX_ Total (<12 years)	1	NA	NA	NA	3.58	−4.03	11.20	0.357
ABC-C_FX_ Stereo (<12 years)	1	NA	NA	NA	0.56	−0.61	1.72	0.351
ABC-C_FX_ Hyper (<12 years)	1	NA	NA	NA	1.17	−1.00	3.33	0.291
SPM-P Soc Part (<12 years)	1	NA	NA	NA	−0.90	−3.99	2.19	0.568
ABC-_FX_ Speech (<12 years)	1	NA	NA	NA	−0.90	−1.39	−0.41	<0.001 *
Vineland-II Mal (>12 years)	1	NA	NA	NA	−0.60	−0.91	−0.29	<0.001 *
VAS Comp (>12 years)	1	NA	NA	NA	2.79	1.68	3.89	<0.001 *
VAS Dis and Anx (>12 years)	1	NA	NA	NA	−14.10	−49.36	21.16	0.433
ABC-C_FX_ Speech (>12 years)	1	NA	NA	NA	0.56	−0.45	1.56	0.277
ADAMS Total (>12 years)	1	NA	NA	NA	−10.63	−13.55	−7.71	<0.001 *
SRS (>12 years)	1	NA	NA	NA	−6.58	−10.28	−2.87	0.001 *
RBS-S (>12 years)	1	NA	NA	NA	−5.35	−7.60	−3.09	<0.001 *
**Performance Based Assessment ^†^**								
MSEL Comp (<12 years)	1	NA	NA	NA	1.47	−1.7	4.64	0.364
ADOS (<12 years)	1	NA	NA	NA	-0.78	−2.88	1.31	0.463
PLS5_ac_rs (<12 years)	1	NA	NA	NA	3.85	−0.12	7.82	0.057
PLS5_ec_rs (<12 years)	1	NA	NA	NA	3.41	−0.55	7.36	0.091
PLS Composite (<12 years)	1	NA	NA	NA	7.26	−0.3	14.82	0.060
RBANS (>12 years)	1	NA	NA	NA	0.69	−2.29	3.67	0.650
Memory 10 Test (>12 years)	1	NA	NA	NA	0.05	−0.02	0.12	0.188
KiTAP 3 module (>12 years)	1	NA	NA	NA	-0.07	−0.18	0.03	0.168
KiTAP go no go (>12 years)	1	NA	NA	NA	-42.70	−145.12	59.72	0.414

**Abbreviations:** FXS, fragile X syndrome; CGI-I, Clinician Global Impression-Improvement; Vineland-II Comp, Vineland Adaptive Behavior Scale-2nd edition, Composite; Vineland-II Mal, Maladaptive Behavior Subscale; ABC-C_FX_ Irr, Aberrant Behavior Checklist-Community Irritability refactored for FXS; ABC-C_FX_ SU/L, Socially Unresponsive/Lethargy; ABC-C_FX_ Hyper, Hyperactivity; ABC-C_FX_ Stereo, Stereotypy; ABC-C_FX_ Speech, Inappropriate Speech; ABC-C_FX_ SA, Social Avoidance; ABC-C_FX_ Total, Total Score; VAS Comp, Visual Analog Scale, Composite; VAS Dis and Anx, Disruptive and Anxiety Behavior Subscales; SPM-P Soc Part, Sensory Processing Measure—Preschool Social Participation Subtest; ADAMS Total, Anxiety Depression and Mood Scale Total Score; SRS, Social Responsiveness Scale™; RBS-S, Repetitive Behavior Scale; MSEL, Mullen Scales of Early Learning-Composite; ADOS, Autism Diagnostic Observation Schedule; PLS-5, Preschool Language Scale, Fifth Ed; ac_rs, auditory comprehension raw score; ec_rs, expressive communication raw score, PLS-5 Comp, Composite Score; RBANS, Repeatable Battery for the Assessment of Neuropsychological Status; KiTAP, the Test of Attentional Performance for Children-3 modules, go/no-go subtests. ^‡^ Meta-Analysis was conducted on subscales that were used in 2 or more papers, using a pre-post correlation of 0.7, ^†^ but not on subscales used in 1 paper. * Indicates p-value < 0.05. ** The ABC-C subscale was used for lethargy and irritability in 2 studies, in contrast to the ABC-CFX subscale used in other studies.

**Table 2:** Placebo-treated groups with FXS in failed clinical trials: substantial placebo effect in endpoint CGI-I.

N Endpoint	Mean Endpoint	Mean Endpoint SEM	Reference
37	3.40	0.17	[23] (>12 yo)
15	3.10	0.27	[23] (<12 yo)
82	3.50	0.14	[26] (>12 yo)
37	3.40	0.17	[27] (>12 yo)
15	3.10	1.25	[27] (<12 yo)
27	2.60	0.16	[28] (<12 yo)
62	3.06	0.11	[29] (>12 yo) *
MD	MD	MD	[30] (>12 yo) **
44	3.30	1.00	[25] (<12 yo) ***
62	3.10	0.12	[25] (>12 yo) ***
ND	ND	ND	[24] (>12 yo) **
25	3.07	MD	[38] (>12 yo)

**Abbreviations:** FXS, fragile X syndrome; CGI-I, Clinician Global Impression-Improvement; N, number; SEM, standard error of the mean; MD, missing data; ND, no data. * Mean endpoint represents mean change. ** Mean endpoint was not reported in the study. *** Mean endpoint was reported, but mean baseline was not reported.

**Table 3:** Outcome measures changes in placebo-treated groups with FXS in failed clinical trials–Sensitivity Analysis.

Endpoint	of Studies	Heterogeneity (I^2^)	Heterogeneity *p-*Value	Meta-Analysis Type	Mean Score Change	95% CI		Change *p*-Value
						Lower	Upper	
**Clinician Rated ^‡^**								
CGI-I (<12 years)	3	0.97	0.00	Random	−0.45	−2.00	1.10	0.570
CGI-I (>12 years)	4	1.00	0.00	Random	0.46	−1.83	2.76	0.693
**Caregiver Rated ^‡^**								
Vineland-II Comp (<12 years)	2	0.00	0.47	Fixed	1.71	0.38	3.05	0.012 *
ABC-CFX Irr (<12 years)	3	0.85	0.00	Random	−3.49	−8.32	1.35	0.157
ABC-CFX SU/L (<12 years)	2	0.97	0.00	Random	−2.28	−7.41	2.85	0.383
ABC-CFX SA (<12 years)	2	0.98	0.00	Random	−1.44	−4.07	1.20	0.284
VAS Comp (<12 years)	2	0.00	0.42	Fixed	1.54	0.90	2.17	0.000 *
Vineland-II Comp (>12 years)	4	0.00	0.44	Fixed	1.74	0.08	3.39	0.040 *
ABC-CFX Total (>12 years)	2	0.00	0.72	Fixed	−8.60	−12.45	−4.76	<0.001 *
ABC-CFX Irr (>12 years) **	4	0.91	0.00	Random	−1.96	−5.11	1.19	0.222
ABC-CFX SU/L (>12 years) **	4	0.93	0.00	Random	−1.90	−4.51	0.71	0.154
ABC-CFX Stereo (>12 years)	2	0.00	0.69	Fixed	−1.04	−1.58	−0.50	<0.001 *
ABC-CFX Hyper (>12 years)	2	0.00	0.84	Fixed	−1.37	−2.26	−0.49	0.002 *
ABC-CFX SA (>12 years)	3	0.79	0.01	Random	−1.65	−2.61	−0.68	0.001 *

**Abbreviations:** FXS, fragile X syndrome; CGI-I, Clinician Global Impression-Improvement; Vineland-II Comp, Vineland Adaptive Behavior Scale-2nd edition, Composite; ABC-CFX Irr, Aberrant Behavior Checklist-Community Irritability refactored for FXS; ABC-CFX SU/L, Socially Unresponsive/Lethargy; ABC-CFX Hyper, Hyperactivity; ABC-CFX Stereo, Stereotypy; ABC-CFX SA, Social Avoidance; ABC-CFX Total, Total Score; VAS Comp, Visual Analog Scale Composite. ^‡^ Meta-Analysis was conducted on subscales that were used in 2 or more papers, using a pre-post correlation of 0.5. Meta-analysis conducted using a pre-post correlation of 0.0 has almost identical values. * Indicates *p*-value < 0.05. ** The ABC-C subscale was used for lethargy and irritability in 2 studies, in contrast to the ABC-CFX subscale used in other studies.

**Table 4:** Children with FXS in placebo-treated failed clinical trials: outcome measures improved.

Endpoint	Correlation	of Studies	Heterogeneity (I^2^)	Heterogeneity *p*-Value	Meta-Analysis Type	Mean Score Change	95% C		Change *p*-Value	% Improvement
							Lower	Upper		
**Caregiver Rated**										
Vineland-II Comp	0.7	2	0.00	0.47	Fixed	1.71	0.38	3.05	0.012	2.51
Vineland-II Comp *	0.5	2	0.00	0.47	Fixed	1.71	0.38	3.05	0.012	2.51
VAS Comp	0.7	2	0.00	0.42	Fixed	1.56	0.94	2.18	<0.001	56.85
VAS Comp *	0.5	2	0.00	0.42	Fixed	1.54	0.90	2.17	<0.001	56.12

**Abbreviations:** FXS, fragile X syndrome; Vineland-II Comp, Vineland Adaptive Behavior Scale-2nd edition, Composite; VAS Comp, Visual Analog Scale, Composite. * Meta-analysis conducted using a pre-post correlation of 0.0 has almost identical values.

**Table 5:** Adolescents/adults with FXS in placebo-treated failed clinical trials: outcome measures improved.

Endpoint	Correlation	of Studies	Heterogeneity (I^2^)	Heterogeneity *p-*Value	Meta-Analysis Type	Mean Score Change	95%CI		Change *p*-Value	% Improvement
							Lower	Upper		
**Caregiver Rated**										ND
Vineland-II Comp	0.7	4	0.00	0.44	Fixed	1.74	0.08	3.39	0.040	ND
Vineland-II Comp *	0.5	4	0.00	0.44	Fixed	1.74	0.08	3.39	0.040	ND
ABC-C_FX_ Total	0.7	2	0.00	0.72	Fixed	−8.60	−12.45	−4.76	<0.001	ND
ABC-C_FX_ Total *	0.5	2	0.00	0.72	Fixed	−8.60	−12.45	−4.76	<0.001	ND
ABC-C_FX_ Hyper	0.7	2	0.00	0.84	Fixed	−1.37	−2.26	−0.49	0.002	ND
ABC-C_FX_ Hyper *	0.5	2	0.00	0.84	Fixed	−1.37	−2.26	−0.49	0.002	ND
ABC-C_FX_ SA	0.7	3	0.79	0.01	Random	−1.65	−2.61	−0.68	0.001	ND
ABC-C_FX_ SA *	0.5	3	0.79	0.01	Random	−1.65	−2.61	−0.68	0.001	ND
ABC-C_FX_ Stereo	0.7	2	0.00	0.69	Fixed	−1.04	−1.58	−0.50	<0.001	ND
ABC-C_FX_ Stereo *	0.5	2	0.00	0.69	Fixed	−1.04	−1.58	−0.50	<0.001	ND

**Abbreviations:** FXS, fragile X syndrome; Vineland-II Comp, Vineland Adaptive Behavior Scale-2nd edition, Composite; ABC-C_FX_ Hyper, Aberrant Behavior Checklist-Community Hyperactivity refactored for FXS; ABC-C_FX_ Stereo, Stereotypy; ABC-C_FX_ SA, Social Avoidance; ABC-C_FX_ Total, Total Score; ND, no data. * Meta-analysis conducted using a pre-post correlation of 0.0 has almost identical values.

**Table 6:** Failed clinical trials in FXS: placebo-treated effect size of improved outcome measures.

Endpoint	N	Baseline Mean Score	Baseline SD	Number of Studies	Pre-Post Correlation	Mean Score Change	Effect Size
Vineland-II Comp (<12 years)	40	68.00	46.57	2	0.7	1.71	0.04
Vineland-II Comp (<12 years) *	40	68.00	46.57	2	0.5	1.71	0.04
VAS Comp (<12 years)	33	2.18	1.47	2	0.7	1.56	1.06
VAS Comp (<12 years) *	33	2.18	1.47	2	0.5	1.54	1.05
ABC-C_FX_ Total (>12 years)	27	44.73	28.01	2	0.7	−8.60	−0.31
ABC-C_FX_ Total (>12 years) *	27	44.73	28.01	2	0.5	−8.60	−0.31

**Abbreviations:** FXS, fragile X syndrome; Vineland-II Comp, Vineland Adaptive Behavior Scale-2nd edition, Composite; VAS Comp, Visual Analog Scale, Composite; ABC-CFX Total, Aberrant Behavior Checklist-Community Total Score refactored for FXS. * Meta-analysis conducted using a pre-post correlation of 0.0 has almost identical values.

## Supplementary Materials

The following are available online at https://www.mdpi.com/2076-3425/10/9/629/s1, Table S1: Randomized-controlled clinical trials in FXS failed to meet primary endpoints.
