# Supplementary material for: Response to Placebo in Fragile X Syndrome Clinical Trials: An Initial Analysis
Source: Brain Sci. 2020 Sep 11;10(9):629. doi: 10.3390/brainsci10090629 (PMC7563217; doi:10.3390/brainsci10090629)
Supplement: Supplementary file 1 [file brainsci-10-00629-s001.pdf]

Supplementary Materials: Response to Placebo in Fragile X Syndrome Clinical Trials: An Initial Analysis

Skylar Luu, Haley Province, Elizabeth Berry-Kravis, Randi Hagerman, David Hessl, Dhananjay Vaidya, Reymundo Lozano, Hilary Rosselot, Craig Erickson, Walter E. Kaufmann and Dejan B. Budimirovic

Table 1. Randomized-controlled clinical trials in FXS failed to meet primary endpoints.

Compound	Primary Endpoint	Phase	Cohort Age*	Sample Size	Duration**	Reference
Mavoglurant	ABC-C$_{FX}$ Total	2b	12-17	139	3	[25]
Mavoglurant	ABC-C$_{FX}$ Total	2b	18-45	175	3	[25]
Arbaclofen	ABC-C Irr	2	6-40	63	4	[22]
Arbaclofen	ABC-C$_{FX}$ SA	3	5-11	172	2	[24]
Arbaclofen	ABC-C$_{FX}$ SA	3	12-50	125	2	[24]
Basimglurant	ADAMS Total	2	14-50	183	3	[28]
Metadoxine	ADHD-RS-IV	2	14-55	62	1.5	[29]
Minocycline	CGI-I; VAS Comp	2	3-16	66	6	[26]
Sertraline	MSEL-EL; CGI-I	2	2-6	52	6	[27]
CX516	Memory Domain	2	18-50	49	1	[23]

Abbreviations: FXS, fragile X syndrome; ABC-C$_{FX}$ Total, Aberrant Behavior Checklist-Community Total Score refactored for FXS; ABC-C$_{FX}$ SA, Social Avoidance; ABC-C Irr, Aberrant Behavior Checklist-Community, Irritability Subscale; ADAMS Total, Anxiety Depression and Mood Scale Total Score; ADHD-RS-IV, Attention Deficit Hyperactivity Disorder Rating Scale-IV; CGI-I, Clinician Global Impression-Improvement; VAS Comp, Visual Analog Scale, Composite; MSEL-EL, Mullen Scales of Early Language-Expressive Language; Memory Domain, Visual Memory and Visual Sequential Memory Subtests of the Test of Visual—Perceptual Skills, Memory for Words Subtest of the Woodcock-Johnson Tests of Cognitive Ability—Revised, Repeatable Battery for the Assessment of the Neuropsychological Status. *In years, **In months.
